# A Group-Based Mobile Application to Increase Adherence in Exercise and Nutrition Programs: A Factorial Design Feasibility Study

**DOI:** 10.2196/mhealth.4900

**Published:** 2016-01-15

**Authors:** Honglu Du, Anusha Venkatakrishnan, Gregory Michael Youngblood, Ashwin Ram, Peter Pirolli

**Affiliations:** ^1^ Palo Alto Research Center Palo Alto, CA United States

**Keywords:** mobile phone, app, social support

## Abstract

**Background:**

Novel methods of promoting self-monitoring and social support are needed to ensure long-term maintenance of behavior change. In this paper, we directly investigate the effects of group support in an exercise and nutrition program delivered by an mHealth application called Fittle.

**Objective:**

Our first specific study aim was to explore whether social support improved adherence in wellness programs. Our second specific study aim was to assess whether media types (ePaper vs mobile) were associated with different levels of compliance and adherence to wellness programs. The third aim was to assess whether the use of an mHealth application led to positive changes to participants’ eating behavior, physical activity, and stress level, compared to traditional paper-based programs.

**Methods:**

A 2 × 2 (eg, Media: Mobile vs ePaper × Group Type: Team vs Solo) factorial design feasibility study was conducted. A sample of 124 volunteers who were interested in improving eating behavior, increasing physical activity, or reducing stress participated in this study. The study duration was 8 weeks. All groups were self-directed with no ongoing human input from the research team.

**Results:**

Participants in ePaper conditions had higher attrition rates compared to participants in Mobile conditions, χ_3_^2^=9.96, *P*=.02 (N=124). Participants in Mobile conditions reported their compliance with a much higher frequency closer to the time of challenge activity completion (2-sample Kolmogorov-Smirnov test comparing distributions was highly significant—KS=0.33, *P*<.001 [N=63]). Participants in ePaper conditions had a much higher frequency of guessing while reporting as compared with those in Mobile conditions—χ_1_^2^=25.25, *P*<.001 (N=63). Together, these findings suggest that the mobile app allowed a more accurate method to report and track health behaviors over a longer period than traditional ePaper-based diaries or log books. There was a significant difference in the overall compliance score for Mobile-Solo (Mean [SD] 0.30 [0.39]) and Mobile-Team (Mean [SD] 0.49 [0.35]) conditions (*t*_50.82_=1.94, *P*=.05). This suggests that working in a team increased participants’ overall compliance within Fittle. Survival analysis showed that participants assigned to Team conditions are 66% more likely to engage longer with mHealth app-based intervention than those assigned to the Solo condition. Overall, participants across all groups reported some positive changes in eating behavior, physical activity, and stress level; however, participants in the Mobile-Solo condition reported higher perceived stress levels at the end of the study.

**Conclusions:**

The team-based Fittle app is an acceptable and feasible wellness behavior change intervention and a full randomized controlled trial to investigate the efficacy of such an intervention is warranted.

##  Introduction

It is no longer news that US health care costs are staggering and that a major driver of these increased costs are unhealthy behaviors such as physical inactivity, increased food intake, and unhealthful food choices [1], which are potentially preventable by health behavior change. In this context, existing formal programs (both primary care and commercial) and one-on-one coaching can be effective in producing behavior change and desirable health outcomes [2]. However, these programs typically have bottlenecks in providing sufficient numbers of expert counselors and personalized day-to-day support to a large population for a long period of time. There is a pressing need to extend the reach of existing health behavior change techniques in areas such as diet and fitness and to intensify and prolong their impact.

Additionally, changing behavior and sustaining it over the long term has been proven to be very difficult and it is suggested that novel methods of promoting self-monitoring and social support are needed to ensure long-term maintenance of behavior change [3]. Social- and group/team-based behavior-change techniques have been shown to be effective in supporting behavior change in areas such as long-term weight loss [4].

Mobile phones may now be providing a platform to scale and disseminate effective group support for sustainable health behavior change [5]. Although eHealth approaches often suffer from the “Law of Attrition” [6] wherein there is a high attrition rate among participants, team identification and interpersonal accountability may ameliorate such attrition. Mobile phones are increasingly prevalent in everyday life and are increasingly used for multiple forms of online social interaction. In this study, we sought to directly investigate the effects of team-based social support in a nutrition and exercise program delivered by an mHealth application called Fittle.

### Social Support in Behavior Change Interventions

Social support may potentially help sustain engagement with health behavior change interventions and consequently increase efficacy. An observational study of more than 80,000 users in the context of a Web-based health promotion intervention [7] revealed that increased social ties within this challenge community directly predicted online engagement and activity completion [8]. In order to systematically characterize the role of social support in enhancing the efficacy of the intervention, a recent study compared a structured physical activity intervention comprising education, activity monitoring, and online social networking via a Facebook group versus an education-only control [9]. Despite the lack of difference between intervention and control groups in physical activity levels post-intervention, the authors did find that the attrition levels for participants in the online intervention group were significantly lower than that of the education-only control group at 12 weeks. Likewise, in another study, it was found that weight loss in a 6-month, remotely delivered weight-loss intervention was strongly associated with engagement within an online Twitter-based social network wherein participants provided each other with informational support [10]. Similar results were found in a Web-based weight-loss program where social networking and personalized recommendations did not demonstrate additive effects for user weight loss or retention, but these features increased the average number of days that a user engaged with the system [11]. More recently, Pechmann et al [12] studied the effectiveness of automatic messages delivered to small online groups via Twitter on abstinence from smoking. They found that specific tweet content about quitting was strongly related to abstinence along with high engagement from users over a period of 100 days.

The concept of social support has also been incorporated into many commercial mobile phone-based health behavior change applications, such as MyFitnessPal, WeightWatchers. In these apps, social support is usually built around the user’s personal social network, such as Facebook or Twitter friends. However, it is not clear whether a social community of this kind is the one best suited for health behavior change. While family and friends might be a good source of emotional support, they might not necessarily have the same level of motivation to engage in health behavior change programs, and they might also not be able to provide the kind of quality informational support that is an important factor in successful behavior change programs [10].

Further, although social support has health benefits in its own right [13] and increases participation in exercise programs [14], building successful groups to support health-related interventions is not automatic. For example, in an effort to test the effects of an online community for helping people to quit smoking, researchers gave 684 people access to an online community in addition to the informational website Smokefree.gov. However, so few people used the online community features that the researchers were not able to report on its effectiveness [15]. There are a few previous studies that aimed to understand the effects of a social network [16] and the potential of a social mobile phone application in improving physical activity level [17]. However, both of these studies focused more on the usability aspects of the behavior change systems and no data on the effects of social support were reported.

In this study, instead of forming social groups from a person’s existing social network, we aim to explore the feasibility of creating an effective behavior-change social group by randomly grouping people who are interested in healthy behavior-change programs into a “team.”

### Mobile Phone-Based Behavior Change Interventions

Mobile phone-based self-monitoring applications have been found to be effective in improving adherence. For example, My Meal Mate (MMM), a mobile weight-loss app developed on Android, allows users to set goals, self-monitor diet and activity, and receive feedback via a weekly text message. In that study, adherence to the mobile phone-based intervention was found to be the highest [18], as compared to paper or Web-based interventions.

However, most of the behavior change content in existing formal programs (both primary care and commercial) and one-on-one coaching are still paper-based. Previous research has shown that a mobile diary was slightly more preferred, but the inability to backfill missing entries resulted in a large prevalence of missing data [19]. However, the sample size of that study was very small (N=12). Thus, a secondary aim of this study was to better understand how the mobile phone-based functionality of an mHealth application differed from a usual-care approach of providing paper-based content and requiring paper journaling. In this study, a PDF version of the program is sent to participants in the paper conditions. They can choose to either view the PDF file on their computers or print it out. In this study, we call it “ePaper” journaling.

Earlier work [20] reported on a mobile phone-based app, Fittle, which includes functionality to promote online group-based support for individuals to progress through behavior-change programs called “challenges.” The challenges are designed to help people master one health-improving habit after another, in a way that builds on previous achievements. Fittle provides a built-in, private social networking feature that allows users to work and engage with their team members so that they can support each other as they learn and adopt new, healthier habits (details about the application are described below). Fittle incorporates a number of evidence-based techniques that are motivated by the theory of planned behavior and social cognitive theory to positively impact health behavior change. Moreover, these interventions can be delivered at low cost because the scaffolding for individual success is either automated or provided through peer support.

The study in Du et al [20] was not designed to systematically study the effects of having team support as all participants were assigned to teams. However, hierarchical regression analyses, including team as a mediating factor, and post-hoc analyses of the impact of team interactions on outcomes were both strongly suggestive of an association between teams, program adherence, and health outcomes. In this current feasibility study, we specifically aimed to compare the Fittle mobile phone-based health behavior change program administered in a solo versus group environment. We hypothesized that using a theory-guided, group/team-based behavior change mobile app (Mobile-Team condition) will result in higher adherence, more improved eating habits, reduced stress, and improved fitness level compared to Mobile-Solo, ePaper-Team, and ePaper-Solo conditions.

### Fittle

**Figure 1:**
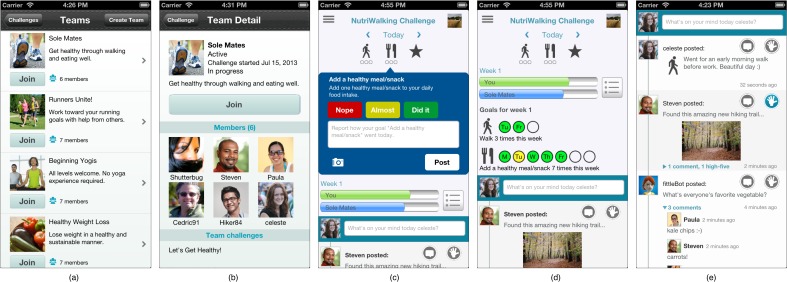
Fittle mobile application screens: (a) teams available, (b) the details of a team, (c) activity information, (d) overall goals for this week, and (e) the team-based social activity feed.

Fittle is implemented as a client-server architecture running on both iOS and Android. Figure 1 shows several screens of Fittle. The initial experience starts with a presentation of a selection of available challenges. These challenges are either conversions of existing programs that have been developed over the years or new programs created by professionals. The app platform is designed to support open content creation by anyone (under some quality control review process). After challenge selection, a user can either join an existing team (Figure 1a) or create a new one and invite teammates via email. The selection of a challenge and membership in a team opens the primary Fittle dashboard (Figure 1c).

### Activities in Fittle

The Fittle dashboard consists of 3 parts. The top portion shows the activities (or goals) as part of the challenge that the user should complete on that given day (ie, today). An icon represents each of these goals with a completion status shown below as a set of circles similar to a horizontal traffic light. Tap selection of the activity icons (as shown in Figure 1c) opens up the title, basic reminder details, the ability to substitute the task for another, a link to more detailed information about the activity that is presented as an electronic card (which may include video demonstration, images, detailed instructions, substitution suggestions, background information, external reference links, links to other related Fittle cards, and more), and the ability to self-report completion and submit a multimedia-enabled post to the team activity feed related to the selected activity. Users can also navigate back and forth in time by tapping the arrows associated with the day display. Users may update reports on past activities, but not on future scheduled activities.

### Progress Tracking

The middle section of the dashboard provides visual analytics showing the user’s and the team’s goal accomplishment for the current week. A details button next to the progress bars opens a weekly activity set view, as illustrated in Figure 1d, showing the user all activities that Fittle will schedule for them in the coming week with visual completion details.

### Team Interaction

An activity-posting bar is always present in the dashboard and provides a means for the user to share multimedia posts with the team at any time (Figure 1e). Text whispers (ie, the faint text that appears in text entry fields before the user enters anything) in all open text fields invite the user to share information with the team. Users may also communicate directly with each other through a peer-to-peer messaging system.

All teams also include an artificial intelligence agent as a member, named “FittleBot.” FittleBot provides daily tips to the team relevant to their activities, previews the activities for the week, and comments on the daily activities of the team members as a group.

### Challenges

In this feasibility study, we featured 2 8-week challenges developed in collaboration with 2 certified experts in personal training and nutrition consulting. These challenges promoted 3 classes of behavior: nutrition, walking, and stress relieving workouts [20].

The challenges consisted of a beginner-level program to get people moving more called “NutriWalking” and a workout focusing on relieving stress called “StressBusting.” Both challenges contained 4 nutrition activities: eat slowly, add a serving of vegetables (or a different vegetable if a vegetarian), add a small healthy meal while reducing the others, and keep a food diary. These activities were offered for 2 to 3 weeks each with overlap in the transition week from one habit to the next.

NutriWalking focused on getting participants to walk more, starting with 15 minutes 3 times a week on flat surfaces and ramping up to 45 minutes 5 times a week on inclined surfaces with some exercises (eg, jumping jacks) or short jogging sessions added to the walk. For off days, stretching was suggested. The system dynamically adjusted a user’s schedule and recommendations if he or she deferred certain activities and/or substituted walking days.

StressBusting focused on 3 scheduled workouts during the week. Mondays comprised an upper body workout consisting of 5 exercises with easier alternatives that focused on strengthening the upper body and core (eg, chest presses, push-ups, high planks, rows). Wednesdays focused on a full body workout consisting of 5 exercises with easier alternatives that focused on muscle endurance (eg, goblet squats, rotation push-ups, jumping rope, mountain climbers, burpees). Finally, Fridays comprised a lower body workout consisting of 5 exercises with easier alternatives that focused on strengthening the lower body and core (eg, deadlifts, squats, supine hip exercises, lunges, ball chops). Tuesdays, Thursdays, and Saturdays were recovery days where participants were asked to engage in a fun and nonstressful activity to get them moving. Sundays were complete rest days. This program was designed on a schedule with the caveat that if a day was missed, it would not be a big deal and there was no need to make it up—users were just asked to keep moving forward without any worry or stress.

### Study Hypotheses

We had 4 research hypotheses (RHs) concerning participants’ compliance, adherence, and the effectiveness of Fittle.

#### RH1: Higher Compliance in Mobile Conditions

The fact that the Fittle app is always available to people on their mobile phones should induce greater compliance. Compliance is defined as the percentage of activities that have been reported as “completed” or “partially completed.” For example, if 100 activities were recommended to a user and 90 of the activities were reported as “completed” or “partially completed,” the compliance rate is 90%.

#### RH2: Higher Compliance in Team Conditions

Participants in team conditions should have higher levels of compliance due to increased social interactions.

#### RH3: Greater Adherence in Team Conditions

Participants in team conditions should have greater adherence to the program due to increased social interactions. Adherence is measured by the number of weeks before a participant becomes inactive (not report any activities for an entire week) in Fittle. For example, if a user always reported some activities from week 1 to week 3, and in week 4 the user did not report any activities, the adherence of this user is 3 weeks.

#### RH4: Superior Outcomes in the Mobile-Team Condition

Participants in the Mobile-Team condition should show the greatest improvements on our 3 outcome measures (eg, stress reduction, improved eating habits, and increased physical activity) due to higher compliance and greater adherence.

## Methods

We designed a 2 × 2 (Mobile vs ePaper × Team vs Solo) factorial design feasibility study to compare the compliance, adherence, and outcomes impact of the team-based (as opposed to solo), mobile app-administered (as opposed to ePaper) health behavior modification challenges.

We will run a 2 × 2 (Mobile vs ePaper × Team vs Solo) analysis of variance (ANOVA) analysis to test RH1 and RH2. For RH3, we will conduct a survival analysis to test the hypothesis that participants assigned to teams (Mobile Team) have greater adherence to the program, controlling for their perceived system usability and attitudes toward the system. To test RH4, we will run a series of 2 × 2 (Media: ePaper vs Mobile × Group Type: Solo vs Team) mixed effects analyses of covariance (ANCOVA). The challenges (StressBusting or NutriWalking) will be modeled as random effects and participants’ attitude toward the Fittle program is modeled as a covariate.

### Recruitment Strategy

As in Heffner et al [21], participants were recruited using Web-based recruitment methods by advertising through Craigslist, Mechanical Turk, Google Adwords, Reddit, and Facebook, separately for the NutriWalking and StressBusting challenges. Advertising material encouraged participants to visit the study information website (NutriWalking or StressBusting study website), which included a brief overview of the study. Individuals interested in the study (challenge) then filled out a screening survey on this website. Individuals were eligible for this study if they: (1) were aged 18 or older, (2) were physically capable of performing the exercises in this study (ie, answered yes to all questions on the Physical Activity Readiness Questionnaire) [22], (3) had either an Apple iPhone or an Android phone, (4) resided in the United States, and (5) were willing to commit the necessary time and effort to the study. Participants who completed the pre- and post-surveys were compensated with a US $20 Amazon gift card.

### Procedure

**Figure 2:**
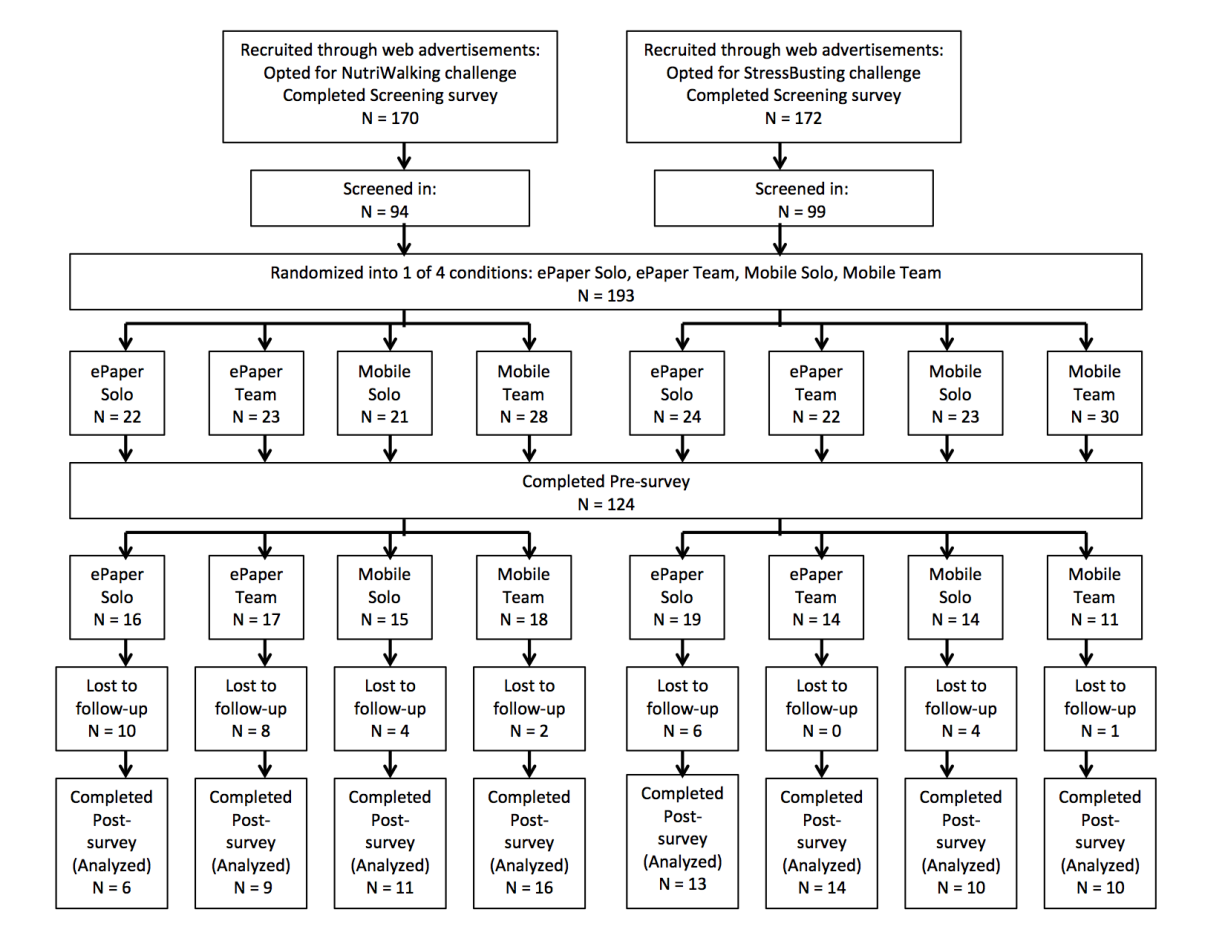
Study flow.

Individuals who passed the initial screening were randomly assigned into 1 of the 4 conditions (eg, ePaper Solo, ePaper Team, Mobile Solo, and Mobile Team) using randomizer.org [23]. Individuals assigned to the ePaper Team and Mobile Team conditions were further randomly assigned into different teams. The teams were created by researchers and were designed to be 12-person groups. Next, participants were emailed a pre-survey. Individuals who completed the pre-survey were included in the study. However, not every individual completed the pre-survey after they were assigned into teams; thus, team sizes ended up being unequal. The final sample consisted of 124 participants (N=66 in NutriWalking; N=58 in StressBusting). Participants were blind to the condition they were in and were not informed that there were different conditions. Figure 2 shows the overall flow of the study.

#### ePaper Solo Condition

Participants in the ePaper Solo (total N=35; N=16 in NutriWalking; N=19 in StressBusting) condition were emailed a PDF version of the wellness program.

#### ePaper Team Condition

Participants in the ePaper Team condition (total N=31; N=17 in NutriWalking; N=14 in StressBusting) were emailed the same PDF version of the wellness program. There were 3 groups in each of the challenges and the final sizes of the groups ranged from 3 to 7 participants. An introductory group email was sent to participants in the same groups and they were encouraged to communicate with each other via email. The groups were then left to their own recognizance.

#### Mobile Solo Condition

There were 29 participants (total N=29; N=15 in NutriWalking; N=14 in StressBusting) assigned to the Mobile Solo condition. Participants were pre-assigned into a 1-person team.

#### Mobile Team Condition

There were 29 participants in the Mobile Team condition (total N=29; N=18 in NutriWalking, N=11 in StressBusting). There were 3 groups in each challenge and the sizes of the groups range from 3 to 9 participants.

For participants in the ePaper conditions, the challenge programs were emailed to them in PDF. Daily activities were listed in the document. The same experts that created the mobile version of the programs prepared this content, which included a daily logging table at the end of the program. Participants were instructed to log their daily compliance and submit the logging table back to the research team at the end of the study.

For participants in the Mobile conditions, the challenge programs were incorporated into the Fittle mobile app. Daily activities were listed on the main page of the Fittle app.

### Data Collected

#### Progress Reports

**Figure 3:**
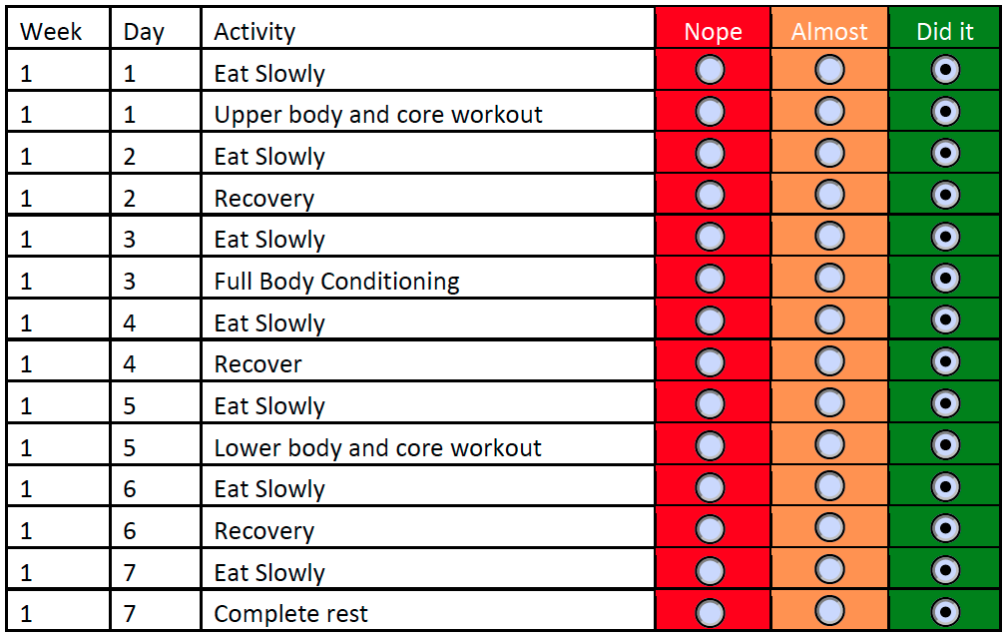
A sample of the logging table in the ePaper conditions.

We collected daily progress reports from participants, allowing us to assess compliance. Participants in the Mobile conditions reported their daily progress within the Fittle app, and participants in the ePaper conditions recorded their daily progress on a logging table (Figure 3) and submitted it to the research team at the end of the study through email or postal mail (2 participants printed the logging table and mailed them to the researchers and the others submitted their reports via email).

#### Presurveys and Postsurveys

Both challenges were designed to help participants improve eating habits, reduce stress, and increase their physical activity level. The following 3 measures were administered in both pre- and post-surveys.

1. Healthy Eating: A 50-item, 5-point scale adopted from Schlundt et al [24] was used to assess participants’ pre- and post-program eating behavior patterns. A lower score indicated healthier eating habits.
2. Perceived Stress Scale: A 10-item, 5-point stress level scale was adapted from Cohen et al [25] to measure participants’ pre- and post-program perceived stress levels. A lower score suggested less perceived stress.
3. Metabolic Equivalent of Task (MET): This was calculated from gender, age, body mass index (calculated from weight and height), resting heart rate, and participants’ usual pattern of daily physical activities as a measure of a person’s cardiorespiratory fitness [26]. A higher value denoted a higher fitness level.

To exclude possible confounds, at post-test we measured perceptions of system usability (for participants in the Mobile Solo and Mobile Team conditions) and the participants’ general attitude toward the program [20].

#### Attitude

A 3-item, 7-point scale (Cronbach’s alpha = .95) was used to measure participants’ general attitude toward the program.

#### Usability

For participants in the mobile condition, a 10-item System Usability Scale (SUS) was used to measure the usability of the Fittle app [27].

Perceived stress scale and SUS scales have all demonstrated good psychometric properties such as discriminant and convergent validity and test-retest reliability in multiple populations [28].

In the post survey, we also asked participants an open-ended question about their experience in the program.

## Results

First, we analyze the baseline characteristics of the participants. We then present analysis of user-generated reports to examine compliance and adherence, followed by pre- and post-test survey results to determine effects on outcomes measures.

Table 1 shows the baseline characteristics of this feasibility study participants by group. Of the 124 participants enrolled, 64.5% (80/124) were female and 86.3% (107/124) were white. The mean age of participants was 36 years (SD=9). A chi-square test was used to determine whether there was a significant difference between ePaper participants and Mobile participants in the education level. As much as 81.82% (54 out of 66) of the participants in the ePaper conditions have at least a college degree, whereas only 63.79% (37 out of 58) of the participants in the Mobile conditions have at least a college degree. This difference was statistically significant, χ_1_^2^=5.14 (N=124), *P*=.03. There were no statistically significant differences found among the groups for the factors balanced at minimization: Gender (*P*=.55) and Age (*P*=.65).

**Table 1:** Characteristics of the participants by conditions.

Characteristics	ePaper Solo (N=35)	ePaper Team (N=31)	Mobile Solo (N=29)	Mobile Team (N=29)
Age in years, mean (SD)	37.63 (8.90)	36.87 (9.0)	35 (8.68)	35.66 (9.85)
Females, n (%)	23 (65.7)	17 (54.8)	19 (65.5)	21 (72.4)
Caucasian, n (%)	31 (88.6)	27 (87.1)	27 (93.1)	22 (75.9)
College degree, n (%)	27 (77.1)	27 (87.1)	17 (58.6)	20 (69.0)
Attrition, n (%)	16 (45.7)	8 (25.8)	8 (27.6)	3 (10.3)
Nonreporters, n (%)	18 (51.4)	9 (29.0)	2 (6.8)	3 (10.3)

*Attrition:* At the end of the study, participants were emailed a post survey. A total of 89 participants responded to the post-survey. Participants (N=35) who did not complete the post-survey were considered dropouts. Table 1 shows the attrition rates in the 4 conditions. Participants in ePaper conditions had higher attrition rates compared to participants in Mobile conditions, χ_3_^2^=9.96 (N=124), *P*=.02. The ePaper Solo condition had the highest attrition rate (45.7%, 16/35), while the Mobile Team condition had the lowest attrition rate (10.3%, 3/29).

*Non-reporters:* Participants who did not report any progress data were considered non-reporters. There were more non-reporters in the ePaper conditions than in the Mobile conditions, χ_3_^2^=21.21 (N=124), *P*<.001. This is not surprising given that it took more effort for people in the ePaper conditions to submit their progress report back to the research team.

### Attitudes Toward the Programs

A 1-way ANOVA with Tukey post-hoc comparisons revealed that participants in the ePaper conditions held more positive attitudes toward the program than participants in the Mobile conditions (*F*_3,46.97_=5.84, *P*=.001, partial η^2^ =.13, Table 2). Specifically, this analysis showed that participants in the ePaper Solo condition reported significantly more positive attitudes (mean [SD] 6.37 [0.87]) than did people in the Mobile Solo condition (mean [SD] 5.05 [1.27], *P*=.01) or marginally significantly more positive than did people in the Mobile Team condition (mean [SD] 5.33 [1.64], *P*=.06).

**Table 2:** Average compliance and attitudes and SD by conditions.

	Mobile Solo	Mobile Team	Paper Solo	Paper Team
Compliance	0.30(0.39)	0.49(0.35)	0.95(0.07)	0.87(0.25)
Attitude	5.05(1.27)	5.33(1.64)	6.37(0.87)	6.04(1.40)

Some insights into this finding were gained by analyzing participants’ responses to the open-ended question about their experience with the program in the post survey. Participants in both the ePaper and Mobile conditions appreciated the progressive nature of the challenges, and found the self-monitoring, bookkeeping part boring, or felt that they did not get enough team support (if they were in a team). Participants in the Mobile condition felt (1) the design of the app was not easy to understand, (2) the challenge team was confusing, (3) did not know how to navigate to different features in the app, and (4) did not feel that they made progress because the app did not give them an easy way to review their progress in the program.

### Social Interactions in Team Conditions

Participants in the Mobile Team condition only interacted with other team members within Fittle while participants in the Paper Team condition used traditional communication channels more often. In the post survey, on a 5-point Likert scale (1: Never, to 5: Every day), participants in both Mobile Team and Paper Team conditions were asked how often they interacted with other team members using email, SMS, or phone calls. Participants in Paper Team conditions (M=2.07, SD=0.94) used these traditional channels more often than those in the Mobile Team conditions (mean [SD] 1.12 [0.22], *t*_23_=4.58, *P*<.001).

Analysis of the server log data showed that on average each participant in the Mobile Team condition interacted with other team members 10 times (SD=14.97), with interactions in the form of posting status updates, comments, or high fives.

### Research Hypotheses Analyses

#### RH1: Higher Compliance in Mobile Conditions

Participants’ average weekly compliance rate was calculated. Average weekly compliance scores were subjected to a 2-way analysis of variance having 2 levels of group types (eg, Solo and Team) and 2 levels of media types (eg, Mobile and ePaper).

The main effect of media type yielded an *F*_1,88_ = 60.82, *P*<.001, indicating that the mean average weekly compliance was significantly greater for participants in ePaper conditions (mean [SD] 0.91 [0.2]) than for those in Mobile conditions (mean [SD] 0.39 [0.38]). The main effect of group type yielded an *F*_1,88_ = 3.24, *P*=.08, indicating that the mean average weekly compliance was not significantly different between Solo (mean [SD] 0.55 [0.44]) and Team conditions (mean [SD] 0.67 [0.46]). The interaction effect was significant, *F*_1,88_ = 4.53, *P*=.04. Participants in the Mobile Team condition reported higher average weekly compliance score than participants in the Mobile Solo condition. However, participants in the ePaper Team condition reported lower average weekly compliance score than participants in the ePaper Solo condition (Table 2).

#### Secondary Analysis on RH1

Given that participant compliance to the Fittle wellness challenge was self-reported, we further probed how accurately participants reported their compliance in both conditions (ie, via Fittle versus ePaper workbooks). In order to do this, we surveyed the responders (ie, completers of the post-survey, N=89). Participants were asked questions in a short follow-up survey to help characterize their reporting behaviors in terms of various time frames relative to challenge activity completion (ie, same day, next day, within 1 week, more than 1 week, and end of study). Participants indicated at which time points they reported/recorded their activity completion, how confident they felt about their report at each of those time points (measured on a 5-point Likert scale, with responses ranging from Very Unsure to Very Sure), and whether they guessed while reporting (measured with a simple yes/no response). Out of 89, N=63 participants completed this survey; N=33 in ePaper and N=30 in Mobile conditions. It is important to note that this survey was specifically agnostic to medium of reporting and did not prime participants in any way about that factor.

**Figure 4:**
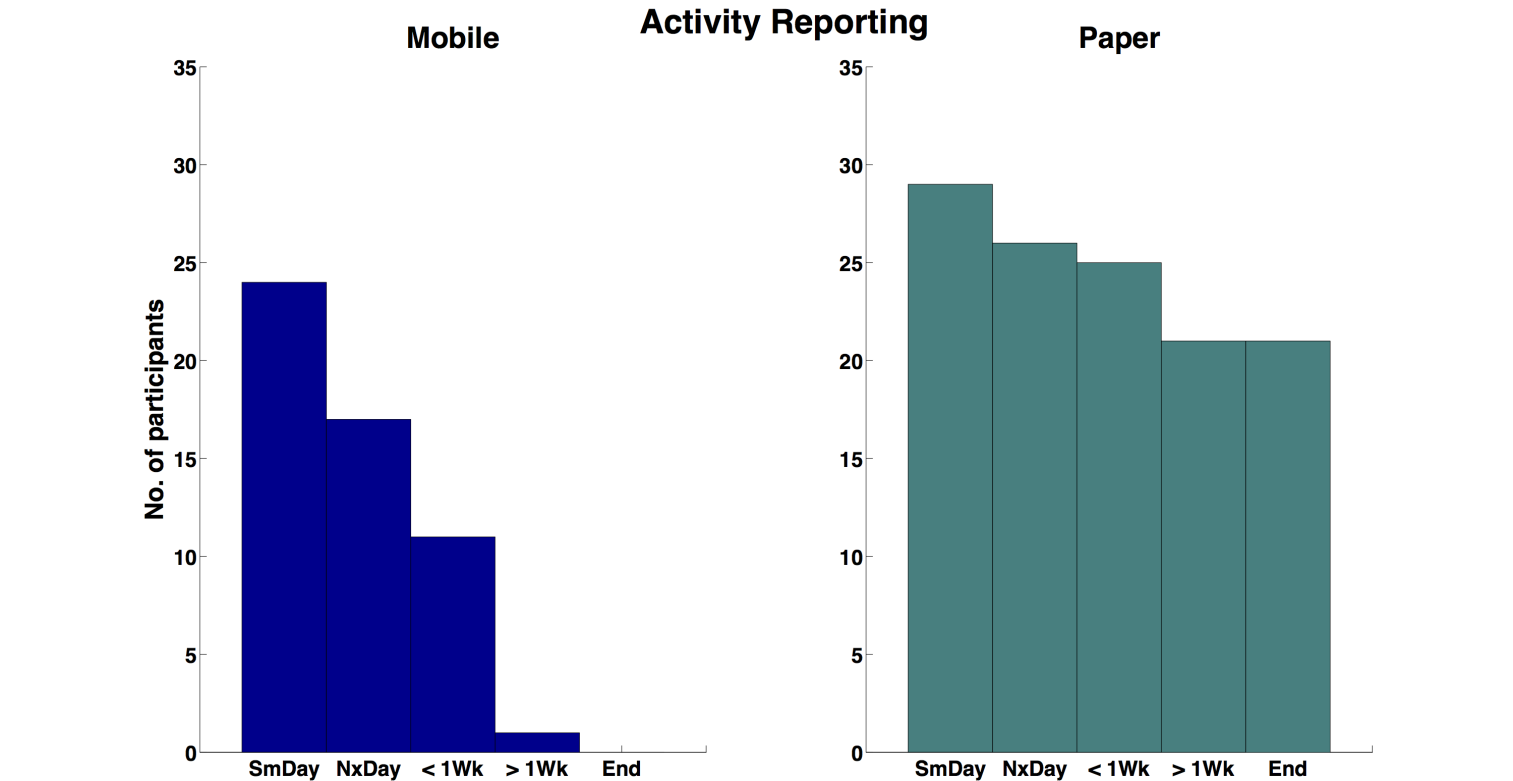
Histograms showing time frequency of participants’ activity compliance self-report in the Mobile and ePaper conditions; frequency distributions were significantly different (Kolmogorov-Smirnov test, P<.001) between Mobile and ePaper conditions. KEY: Sm Day: Same Day as scheduled activity; Nx Day: Next Day after scheduled activity.

As seen in Figure 4, a clear dissociation between participants in the Mobile and ePaper conditions emerged, as participants in Mobile conditions reported their compliance with a much higher frequency closer to the time of challenge activity completion (2-sample Kolmogorov-Smirnov (KS) test comparing distributions was highly significant; KS (N=63) = 0.3254, *P*<.001). On the contrary, participants in the ePaper condition appeared to report their compliance with a constant frequency regardless of temporal proximity to challenge activity completion.

**Figure 5:**
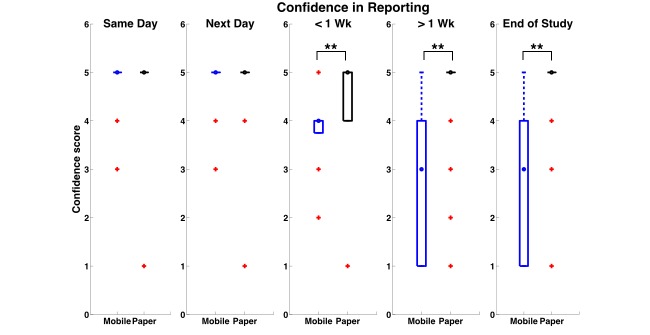
Boxplots showing that confidence in self-report significantly decreased for participants in Mobile condition (Blue) compared to ones in ePaper condition (Black) when self-reporting occurred further away in time with respect to activity occurrence: < 1 week (*P* < .001), > 1 week (*P*< .001) and End of study (*P*< .001). Circles represent medians, boxes represent interquartile intervals and + (Red) represent outliers.

Further, when probed about their confidence in their own compliance report, participants in the Mobile condition again clearly differentiated themselves from those in the ePaper condition (Figure 5). Their confidence progressively decreased, as the time of reporting grew distant from the challenge activity completion time point, which could be indicative of honesty in their reporting behaviors. In contrast, participants in the ePaper condition appeared to have relatively high confidence throughout all time points of reporting regardless of when challenge activity was completed. A Kruskal-Wallis test comparing medians at each of the 5 time points (α=0.01 adjusted for multiple comparisons) was highly significant for the later time points, namely less than 1 week, χ_1_^2^ = 13.14 (N=63), *P*<.001); more than 1 week, χ_1_^2^ = 12.64 (N=63), *P*<.001); and end of study, χ_1_^2^ = 10.99 (N=63), *P*<.001.

**Figure 6:**
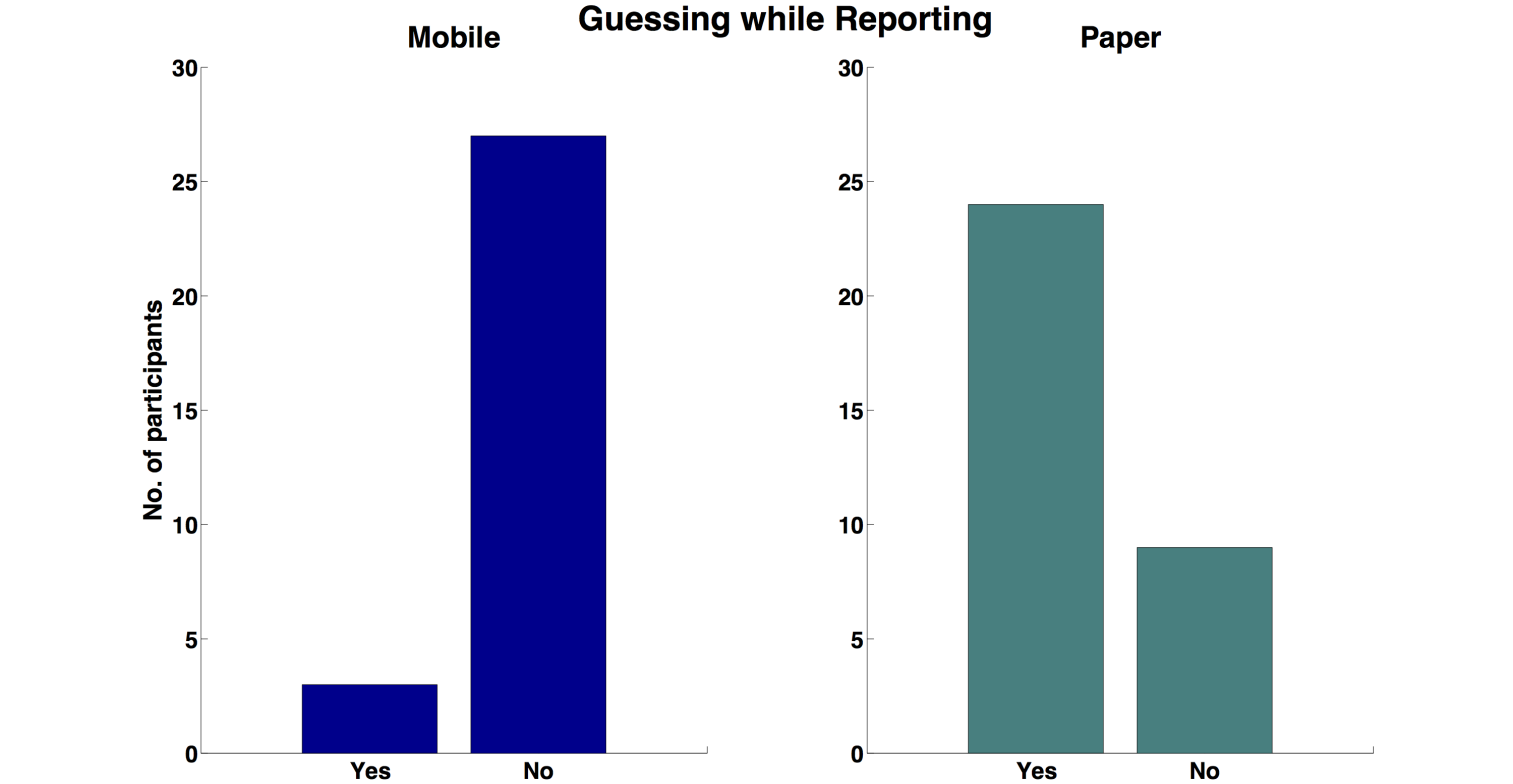
Frequency of guessing during activity compliance reporting.

Finally, when participants were probed about whether they guessed while they reported compliance, the division between participants in the Mobile and ePaper conditions was further solidified (Figure 6). Participants in the ePaper condition had a much higher frequency of guessing while reporting as compared to those in the Mobile condition (chi-square test was highly significant, χ_1_^2^ = 25.25 (N=63), *P*<.001.

#### RH2: Higher Compliance in Team Conditions

Given that the accuracy of self-reporting compliance in the ePaper conditions was much lower than in the Mobile conditions, we subsequently focused on analyzing compliance and adherence from the Mobile conditions only. To understand whether being in a team induced higher compliance than being alone, we compared Mobile Solo vs Mobile Team conditions. We first examined the overall compliance between the 2 conditions. Overall compliance was defined as the average compliance across 8 weeks (ie, challenge duration). There was a significant difference in the overall compliance score for Mobile Solo (mean [SD] 0.30 [0.39]) and Mobile Team (mean [SD] 0.49 [0.35]) conditions (*t*_50.82_=1.94, *P*=.05). This suggests that working in a team increased participants’ overall compliance within Fittle.

**Figure 7:**
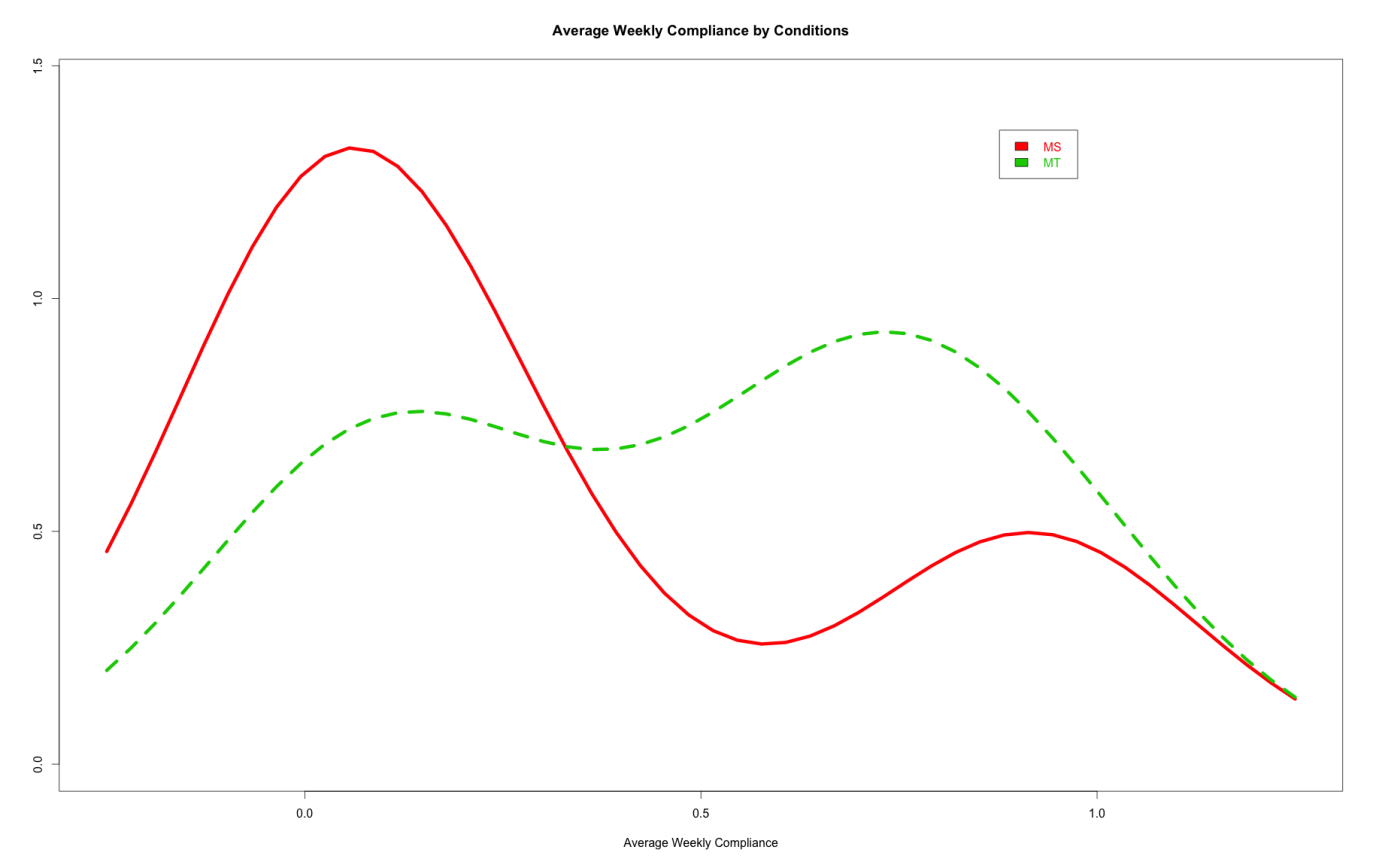
Kernel Density plots of average weekly compliance showing a bimodal distribution for both Mobile-Solo (Red) and Mobile-Team (Green) conditions.

We further examined the distribution of the overall compliance in Mobile Solo and Mobile Team conditions. A kernel density plot (Figure 7) showed that overall compliance in both Mobile Solo and Mobile Team conditions interestingly follows a bimodal distribution. Most participants in the Mobile Solo condition had a very low overall compliance (left modal) and very few participants in the Mobile Solo condition had a high overall compliance (right modal). However, in the Mobile Team condition, the proportion of participants whose overall compliance was around the left modal was reduced and most participants’ overall compliance was around 0.75 (right modal). This dichotomy in the distribution of overall compliance across Team versus Solo conditions suggests that the team social support-based intervention is more likely to shift individual user compliance closer to the team mean (ie, increase individual compliance). The challenge therefore to design more effective socially based health behavior interventions is to focus on increasing overall mean compliance in teams/groups of participants.

#### RH3: Greater Adherence in Team Conditions

Based on the self-reported compliance data, adherence of the ePaper groups was high. The average adherence rate of the ePaper Solo group was 0.97 and ePaper Team group was 0.85 (Table 2). However, given the low confidence in the compliance report in the ePaper conditions, we chose to focus the analysis on the Mobile conditions.

We applied survival analysis to test the hypothesis that participants assigned to teams (Mobile Team) have greater adherence to the program, controlling for perceived system usability and attitudes toward the system. In the present study, the event of interest is the time at which a user disengaged from Fittle. This analysis predicted the length of engagement in Fittle based on whether users worked in teams and other control variables.

We considered a participant to have disengaged if they stopped reporting compliance data for an entire week (ie, the compliance of a week is zero). Participants who were engaged until the end of the 8-week study were treated as right censored in the survival analysis.

**Figure 8:**
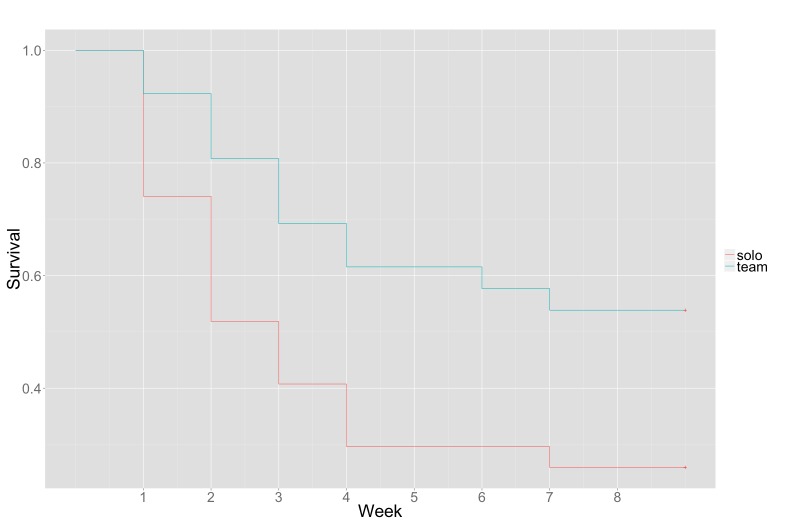
Survival curves for participants in Mobile-Team (Green) and Mobile-Solo (Red) conditions showing a significantly higher survival rate (ie, “adherence” in the Mobile-Team condition; *P*=.02).

Table 3 and Figure 8 show the results of the survival analysis. Effects are reported in terms of the hazard ratio. The hazard ratio value for TeamCondition means that participants assigned to Team condition are 66% more likely to engage longer than those assigned to the Solo condition (100% – [100% × 0.34]). The hazard ratio for Attitude indicates that survival rates are 32% higher for those who had a positive Attitude at least 1 standard deviation greater than average, when all other variables were at their average levels. These findings taken together indicate that the team-based intervention, when combined with a positive attitude toward the mobile phone-based program, can positively impact and increase long-term adherence to the program.

**Table 3:** Results of the survival analysis.

Variables	Hazard ratio	Lower 95% CI	Upper 95% CI
TeamCondition^a^ (0=Solo, 1=Team)	0.34 (*P*=.02)	0.14	0.86
Attitude^b^	0.68(*P*=.02)	0.49	0.95

^a^TeamCondition is a binary predictor variable that describes whether a user was assigned to a Team condition or a Solo condition.

^b^SUS and Attitude are included as covariates to check whether participants’ perceived usability of Fittle and their attitude toward the program interfered with the ability to perform and report compliance data.

#### RH4: Superior Outcomes in the Mobile-Team Condition

Table 4 shows participants’ average pre- and post-changes (measured by (post-pre)/pre) on Healthy Eating, Perceived Stress Scale, and MET. On average, participants experienced changes in the positive directions (ie, healthier eating habits, lower stress levels, and more physically active) except for participants in the Mobile Solo condition. Participants in the Mobile Solo condition reported higher perceived stress levels at the end of the study.

**Table 4:** Mean scores of percentage changes in Healthy Eating, Perceived Stress Scale, and MET, and the significance of 2 × 2 ANCOVAs.

	ePaper		Mobile		Probabilities			
	Solo	Team	Solo	Team	Media	TeamType	Attitude	Education
Healthy eating (%)	-0.15 (0.08)	-0.13 (0.11)	-0.06 (0.10)	-0.05 (0.12)	.001	.62	.002	.30
Perceived Stress Scale (%)	-0.27 (0.23)	-0.20 (0.22)	0.18 (1.05)	-0.08 (0.35)	.02	.39	.006	.35
MET (%)	0.04 (0.17)	0.02 (0.09)	0.04 (0.15)	0.05 (0.19)	.59	.85	.04	.38

In order to assess the relationship between media type, team type, and participants’ improvements in Healthy Eating, Perceived Stress Scale, and MET, a series of 2 × 2 (Media: ePaper vs Mobile × Group Type: Solo vs Team) mixed effects ANCOVAs were conducted, with a modified *P*-value of .017 (Bonferroni correction). The challenges (StressBusting or NutriWalking) are modeled as random effects and participants’ attitude toward the Fittle program and their education level are modeled as covariates.

RH4 was not supported in this study. Table 4 shows the results of the analysis. The influence of Media and TeamType was found to be statistically nonsignificant on participants’ changes in Perceived Stress Scale and MET. In terms of participants’ improvement in eating patterns (Healthy Eating), participants in ePaper conditions reported more improvements (mean [SD] -0.14 [0.09]) than participants in Mobile conditions (mean [SD] -0.06 [0.11]), which was statistically significant. The strength of this relationship, as indexed by η^2^, was .27. Overall, these results suggest that the ePaper-based intervention outperformed the mobile delivery of the intervention in helping participants achieve the health outcomes in this feasibility study.

##  Discussion

### Principal Results

In this project, we studied a novel, low-cost, group-based mobile phone health and wellness application called Fittle. The intervention combined the traditional daily activities suggestions/prompts with real-time, peer-to-peer social support that encouraged discussions consistent with guidelines for behavior change for health and wellness [29,30,31] and online community building [10,12]. More importantly, this application encouraged and helped users to learn and master new, healthier habits progressively over the 8-week period.

Our first specific study aim was to explore if social support improved adherence. Participants in the Mobile Team condition reported higher compliance to the program compared to those in the Mobile Solo condition. Our survival analysis showed that participants assigned to the Team condition are 66% more likely to engage longer than those assigned to the Solo condition. Furthermore, participants’ overall attitude toward the program had a significant effect on participants’ adherence, which supports the notion that efforts to enhance positive attitudes toward these health behavior change programs can greatly increase their impact.

Our second specific study aim was to assess whether media types (ePaper vs Mobile) were associated with different levels of compliance and adherence to wellness programs. We found that participants in the ePaper conditions reported a much higher compliance score. However, the confidence in the compliance report in the ePaper condition was much lower than in the Mobile condition, while the attrition rate was higher in the ePaper condition. One major factor that could have led to this behavioral discrepancy between the 2 conditions is that the affordance of the Mobile app is much lower to support back-reporting in time, which is not the case with the ePaper workbook. This could have led to possible over-reporting in the ePaper condition precisely due to the easy access of the entire ePaper workbook.

These findings are consistent with those of Stone and colleagues [32] who also found that patients maintaining ePaper medication diaries showed a higher frequency of back-reporting or “hoarding.” These results may provide additional support for the use of mobile diaries for patients/users wherein closer tracking of long-term behaviors are required. It also highlights the need for objective measurement of adherence to behavioral treatment regimens, which in the context of physical activity interventions can potentially be achieved with wearable activity trackers.

The third aim was to assess whether the use of Fittle led to positive changes to participants’ eating behaviors, physical activity, and stress level. The results revealed that participants all reported some positive changes in Healthy Eating, Perceived Stress Scale, and MET, except that participants in the Mobile Solo condition reported higher Perceived Stress Scale at the end of the study. No significant effects of media or team type were found for the changes observed in Perceived Stress Scale and MET. However, as for Healthy Eating, participants in ePaper conditions reported more improvements than participants in Mobile conditions. The reason for these results is unclear, and it is not explainable given our study design. One possibility is that the higher attrition in the ePaper groups could have been related to less motivated participants dropping out, leaving a more-motivated ePaper cohort to participate for the study duration. On the contrary, the mobile app may have retained a less-motivated cohort who would have otherwise had less compliance and, consequently, these less-motivated participants did not see the expected health benefits.

### Limitations

Generalizability of the initial findings from this feasibility study is limited given that the sample was mostly white and female. Moreover, recruitment through online advertisements resulted in a selection bias with more highly educated people being involved in the study, which is in line with the results from previous reviews indicating that mainly higher educated individuals participate in online interventions [33]. This further precludes the generalizability of our study results.

The overall attrition rate of this study was 28.2% (35/124). Missing data may indicate a participant’s dissatisfaction with the program. In this study, attrition was not equal among the conditions, with attrition in the ePaper conditions being higher than the Mobile conditions. The attrition rate in the ePaper conditions was about 36.4% (24/66). However, in the mobile conditions (Mobile Solo and Mobile Team), the attrition rate was 18.9% (11/58), which is in line with results from recent reviews that indicated that the average attrition rate in Internet-based physical activity interventions is about 20% [34,35]. However, this is much higher than the 7% attrition rate reported in mobile phone-based interventions [18]. This higher attrition rate may be due to the fact that the Fittle app was a work-in-progress app. Participants held more positive attitudes toward the ePaper-based program than the app-based program. This may have affected participant engagement. Currently, we are working on redesigning these components to improve user experience and accessibility of the health tips-based electronic cards, which could help increase participant engagement in future interventions.

Due to the higher than expected attrition rate, the absolute sample size at the post survey was low, especially for the self-reported compliance, Healthy Eating, Perceived Stress Scale, and MET data. This reduced statistical power could have also partly precluded detection of any intervention effects in the post survey on self-reported Healthy Eating, Perceived Stress Scale, and MET data.

Finally, our initial research design strived to create equal-sized teams. However, not every participant who qualified to participate actually joined the study and the teams ended up having different sizes. In a future clinical trial, the design would need to be altered to address the team size issue and to potentially include analytical approaches to stratify and weight teams based on size while examining intervention efficacy.

### Implications and Future Research

The results from this study provide additional evidence to support the role of positive social/group effect on participants’ adherence in mobile phone-based wellness programs. The sizes of the teams range from 3 to 9 participants. However due to the small number of teams, we did not get much insight into the optimal size of the teams. Future studies on the effects of team sizes on adherence and best practices to organize effective teams are necessary.

Moreover, it should be noted that the Mobile conditions received different forms of social interactions and support (ie, interactions with other participants in Fittle, FittleBot-provided daily tips). However, our study design does not allow us to determine whether all components are equally effective and whether their combination is necessary. Future studies should separate the different intervention components in order to assess their individual impact. Furthermore, recent research suggests that prompts via auto-messages to stimulate social interactions within groups helps significantly increase participation in socially based health behavior change interventions [12,36]. Therefore, further research is necessary to help design more targeted prompts within our mobile phone application to most effectively increase participant engagement.

### Conclusions

In conclusion, in this initial study examining effectiveness of a group-based mobile phone wellness program, we demonstrated that having people work in teams led to a significantly higher level of adherence and engagement over time. Positive changes in participants’ eating patterns, perceived stress, and physical activity levels were reported. We believe that these findings are very promising and should encourage future research to investigate and characterize the role of objective measures of participants’ adherence and social support in mobile phone-based wellness programs.

## Abbreviations

ANOVA: analysis of variance
ANCOVA: analysis of covariance
CI: confidence interval
MET: metabolic equivalent of task
RH: research hypotheses
SUS: System Usability Scale
